# Adult-Onset Still’s Disease Presenting With Leukopenia and Pulmonary Infiltrates: A Mimicker of Infection and Malignancy

**DOI:** 10.7759/cureus.96507

**Published:** 2025-11-10

**Authors:** Zahra Vaezi, Afshin Amini

**Affiliations:** 1 Internal Medicine, St. Luke's Hospital, Chesterfield, USA

**Keywords:** adult-onset still’s disease, diffuse lymphadenopathy, fever of unknown origin (fuo), hyperferritinemia, salmon colored rash

## Abstract

Adult-onset Still’s disease (AOSD) is a rare systemic inflammatory disorder that often mimics infections, tickborne diseases, or malignancies, leading to diagnostic delays. We present a 26-year-old male with persistent fever, arthralgia, lymphadenopathy, and elevated inflammatory markers unresponsive to antibiotics. Extensive infectious and autoimmune workup was largely negative except for positive antinuclear antibody (ANA) and ribonucleoprotein (RNP). Development of a maculopapular rash with spiking fevers and elevated ferritin (1240 ng/mL) met Yamaguchi’s criteria for AOSD. Initiation of corticosteroids resulted in the rapid resolution of fever. This case highlights that AOSD should remain a diagnostic consideration in fever of unknown origin, even with positive autoantibodies.

## Introduction

Adult-onset Still’s disease (AOSD) is a rare systemic autoinflammatory disorder characterized by quotidian fever, evanescent rash, arthritis, and multi-organ inflammation [1,2]. The estimated incidence is 0.16-0.4 per 100,000, with peaks in young adults and middle-aged patients. Diagnosis remains challenging due to nonspecific clinical features and the need to exclude infections, malignancies, and autoimmune diseases such as systemic lupus erythematosus and mixed connective tissue disease (MCTD) [1,3].

The pathogenesis involves dysregulated cytokine activation - especially IL-1β, IL-6, and IL-18 [4-6]. Despite the development of emerging biomarkers such as IL-18 and S100 proteins, Yamaguchi’s criteria remain the most widely accepted diagnostic framework [7-10]. Here, we describe an atypical case of AOSD in a young male patient with pleural effusions and strong antinuclear antibody (ANA) and anti-ribonucleoprotein (RNP) positivity - features that overlap with MCTD - highlighting the diagnostic complexity and the rationale behind the final diagnosis.

## Case presentation

**Figure 1:**
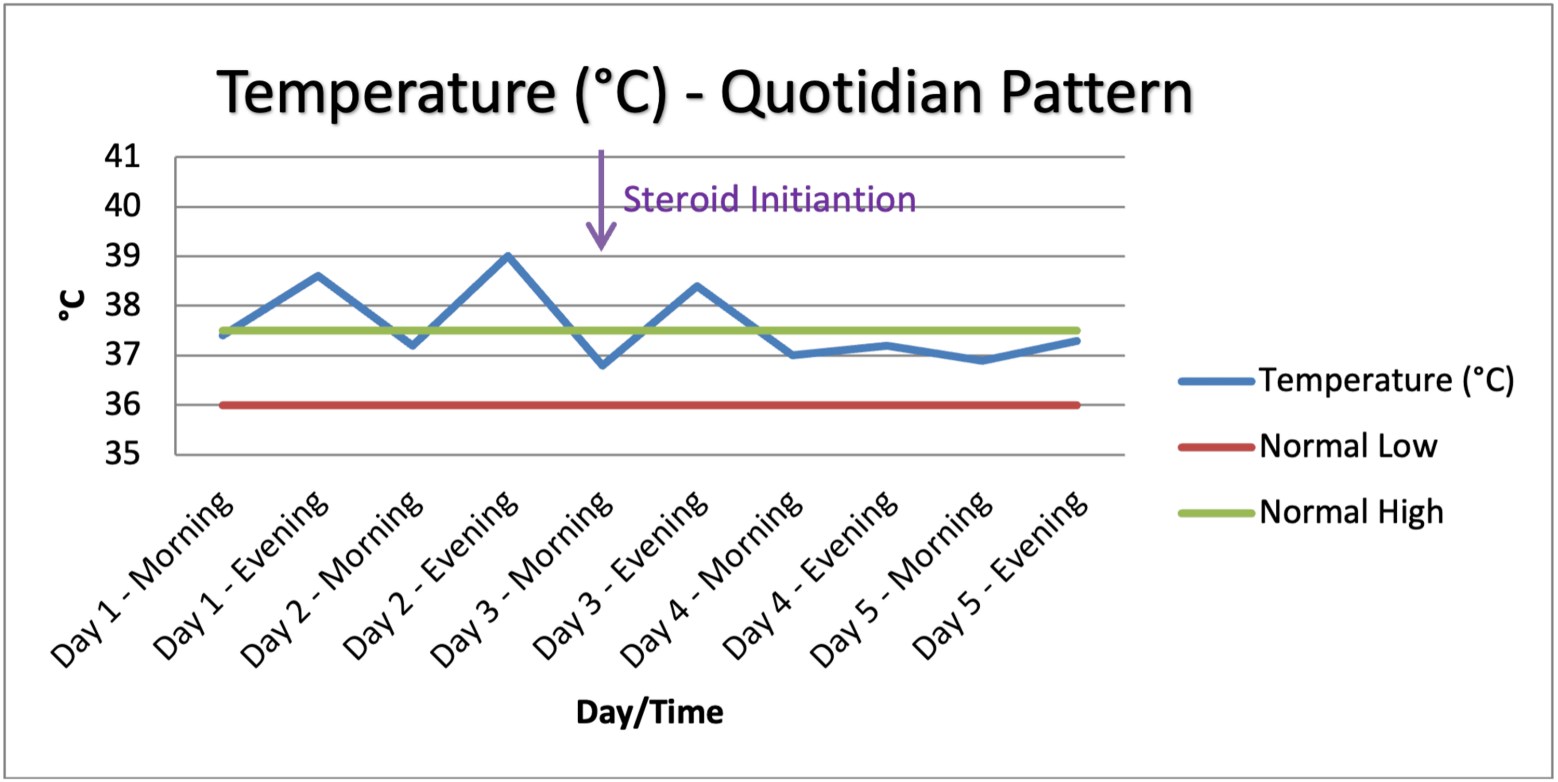
Temperature trend showing fever resolution after day three upon starting systemic corticosteroid therapy

A 26-year-old previously healthy male presented with a two-week history of quotidian fever (peaking daily in the evening) (Figure 1), vomiting, generalized weakness, and diffuse arthralgia. He denied sore throat, chest pain, cough, dyspnea, rash, or recent sick contacts. There was no history of alcohol, tobacco, or intravenous drug use. Outpatient treatment with amoxicillin-clavulanate and levofloxacin had been ineffective.

**Figure 2:**
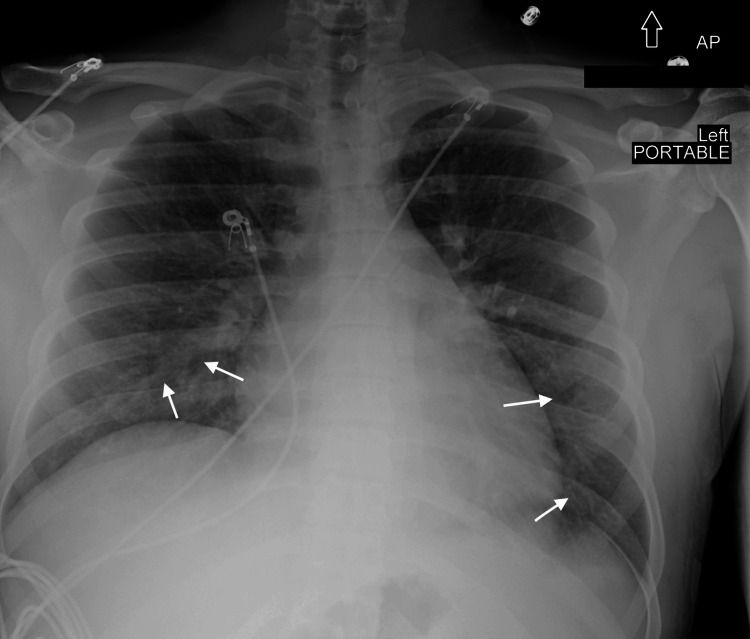
Anteroposterior chest X-ray showing bilateral patchy infiltrates (white arrows)

On arrival to the emergency department, he was tachypneic with a respiratory rate of 25 breaths/min and febrile with a temperature of 39.6 °C. Physical examination revealed diffuse joint tenderness without effusion, no lymphadenopathy, no pharyngeal erythema, and clear lung fields on auscultation. Initial evaluation raised concern for pneumonia based on chest radiography (Figure 2), but he had no significant past medical history or comorbidities.

**Figure 3:**
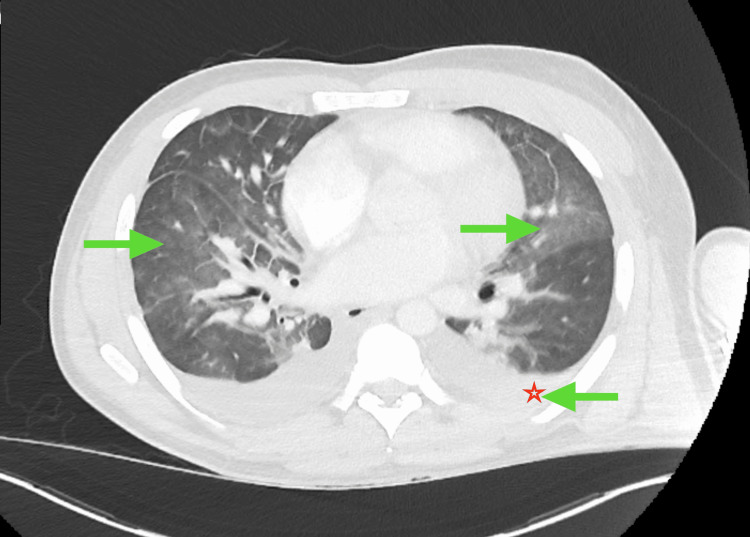
Chest CT scan with and without contrast, axial view, showing bilateral pulmonary infiltration (green arrows) and pleural effusion (green arrow and star)

**Figure 4:**
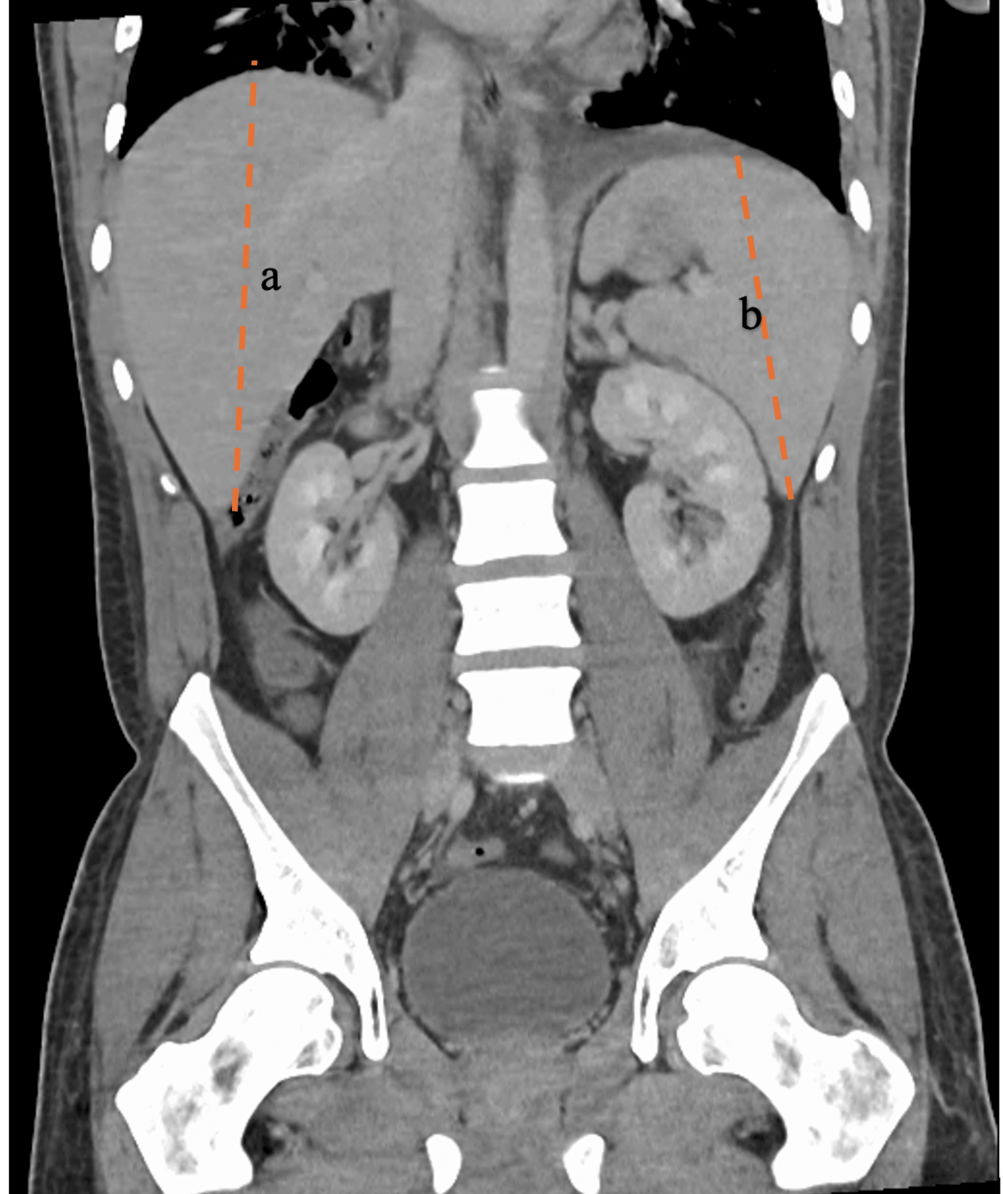
CT scan of abdomen, coronal view, showing hepatomegaly (a, 188 mm), splenomegaly (b, 154 mm)

**Figure 5:**
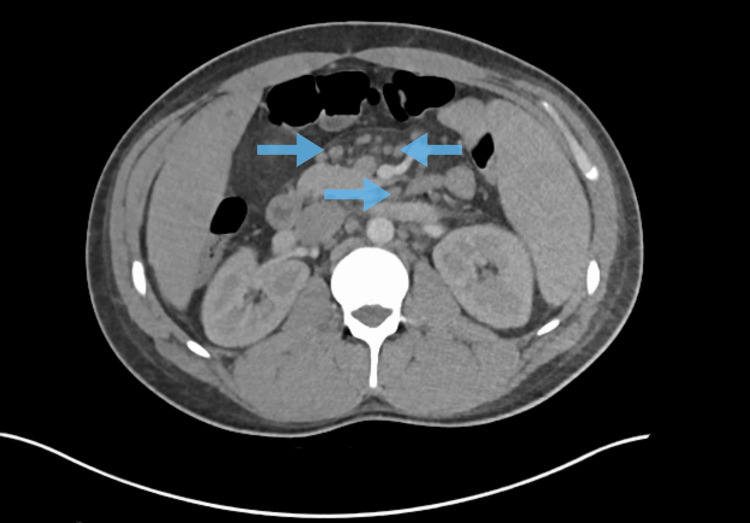
CT abdomen, axial view, showing retroperitoneal lymphadenopathy (blue arrows)

Laboratory investigations, including complete blood count (CBC), comprehensive metabolic panel (CMP), urinalysis, and erythrocyte sedimentation rate (ESR), were largely unremarkable except for leukopenia (white blood cell count 3.2 ×10^3/µL) and a mildly elevated procalcitonin (0.35 ng/mL). Inflammatory markers were significantly elevated: C-reactive protein (CRP) 22.3 mg/dL, lactate dehydrogenase (LDH) 664 U/L, and ferritin 1240 ng/mL. Computed tomography (CT) of the chest demonstrated bilateral pulmonary infiltrates, interstitial edema, and pleural effusion (Figure 3), while CT of the abdomen revealed hepatosplenomegaly (Figure 4) and diffuse lymphadenopathy (Figure 5). The differential diagnosis included hematologic malignancy, metastatic disease, systemic inflammatory conditions, and infectious etiologies.

Despite initiation of broad-spectrum antimicrobial therapy with piperacillin-tazobactam, linezolid, and doxycycline, the patient continued to have quotidian fevers. An extensive infectious workup was negative (Table 1). Rheumatologic testing was unrevealing except for positive ANA and RNP antibody (Table 2).

**Table 1:** Laboratory test results: Infectious work-up * Respiratory panel detects common viral pathogens including influenza A/B, respiratory syncytial virus (RSV) A/B, adenovirus, parainfluenza 1–4, human metapneumovirus, rhinovirus/enterovirus, and seasonal coronaviruses (229E, NL63, OC43, HKU1). Many panels also include SARS-CoV-2 and atypical bacteria such as Mycoplasma pneumoniae and Chlamydophila pneumoniae. **GI PCR panel screens for common bacterial (e.g., Salmonella, Shigella/EIEC, Campylobacter, C. difficile, STEC, Vibrio, Yersinia, ETEC, EPEC, EAEC, Plesiomonas), viral (Norovirus, Rotavirus A, Adenovirus F40/41, Astrovirus, Sapovirus), and parasitic pathogens (Giardia lamblia, Cryptosporidium, Entamoeba histolytica, Cyclospora). GI PCR: Gastrointestinal Polymerase Chain Reaction Panel; CMV: Cytomegalovirus; EBV: Epstein–Barr Virus; HIV: Human Immunodeficiency Virus; Ab: Antibody; Ag: Antigen

Lab Test	Result	Reference range
Blood Cultures-1	Negative	No growth
Blood Culture-2	Negative	No growth
Respiratory Culture	Negative	No growth
Respiratory Panel *	Not Detected	Not Detected
GI PCR **	Not detected	Not Detected
CMV Antibodies (IgM, IgG)	Negative	N/A
EBV Panel	Negative	N/A
HIV Panel	Negative	N/A
Legionella Antigen	negative	N/A
Strep Pneumonia Antigen	Negative	N/A
Ehrlichiosis Antigen	Negative	N/A
Cryptococcal Antigen	Negative	N/A
Lyme Serology	Negative	N/A
Rocky Mountain Spotted Fever Ab	negative	N/A
Histoplasma Galactomannan Ag	Negative	N/A
Mycoplasma Ab	Negative	N/A

**Table 2:** Labratory test results: Immunologic and inflammatory markers *ANCA panel includes indirect immunofluorescence for c-ANCA and p-ANCA patterns, along with antigen-specific enzyme-linked immunosorbent assay (ELISA) testing for PR3 (Proteinase 3) and MPO (Myeloperoxidase) antibodies. ANA: Antinuclear Antibody; Anti-CCP: Anti-Cyclic Citrullinated Peptide; RNP Ab: Ribonucleoprotein Antibody; RF: Rheumatoid Factor; dsDNA Ab: Double-Stranded DNA Antibody; SS-A/SS-B Ab: Sjögren’s Syndrome-Related Antibodies A/B; Sm Ab: Smith Antibody;  ANCA: Anti-Neutrophil Cytoplasmic Antibody; Ferritin: Ferritin (Acute Phase Reactant); CRP: C-Reactive Protein; LDH: Lactate Dehydrogenase; ACE: Angiotensin-Converting Enzyme; Anti-MPO Ab: Anti-Myeloperoxidase Antibody; Anti-PR3 Ab: Anti-Proteinase 3 Antibody

Lab Test	Result	Reference Range
ANA	1:1280 - Speckled Pattern	Negative
Anti CCP-Ag	4	0-19 unit(s)
RNP Ab	146 (H)	<20 U
RF	<9	<15 IU/mL
DNA (DS) Ab	<1	1-19 unit(s)
SS-A Ab	<0.2	0.0-0.9 unit(s)
SS-B Ab	<0.2	0.0-0.9 unit(s)
SM Ab	0.3	0.0-0.9 unit(s)
Ferritin	1240 ng/mL (H)	30–400 ng/mL
CRP	12.1 mg/dL (H)	<0.5 mg/dL
LDH	664 U/L	100-250 U/L
ACE	58 U/L	14-82 U/L
Anti-centromere Ab	<0.2	0.0-0.9 unit(s)
Anti-Myeloperoxidase Ab	<0.2	0.0-0.9 unit(s)
Anti-Proteinase 3 Ab	<0.2	0.0-0.9 unit(s)
Anti Smooth Muscle Ab	0.3	0.0-0.9 unit(s)

**Figure 6:**
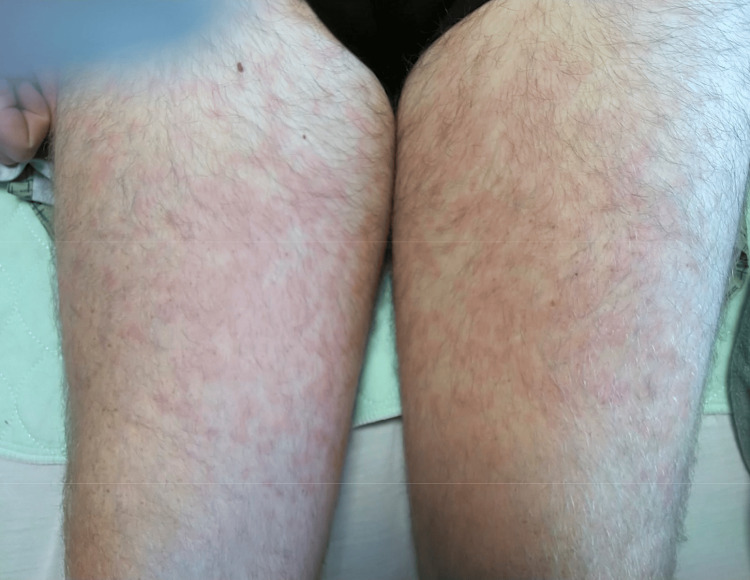
Evanescent salmon-colored maculopapular rash on the patient’s extremities during febrile episodes.

During hospitalization, the patient developed a salmon-colored maculopapular non-itchy rash coinciding with febrile spikes, involving both upper and lower extremities, including the palms and soles (Figure 6).

A retroperitoneal lymph node biopsy demonstrated benign lymphoid tissue with sinus histiocytosis, and flow cytometry excluded lymphoproliferative disease. Given the constellation of quotidian fever, evanescent rash, lymphadenopathy, splenomegaly, leukopenia, hyperferritinemia, and exclusion of infectious and malignant processes, a presumptive diagnosis of AOSD was made.

The patient was initiated on intravenous methylprednisolone 60 mg daily, and later transitioned to oral prednisone 60 mg at discharge. His fevers resolved within four days of corticosteroid therapy, with marked clinical improvement. He was discharged in stable condition and referred for outpatient rheumatology follow-up for ongoing management and consideration of steroid-sparing therapy.

## Discussion

This case illustrates the heterogeneity of AOSD and the diagnostic delays associated with atypical or overlapping features. Clinical presentation varies from monophasic, self-limited disease to chronic, relapsing systemic or articular courses [1-3]. Our patient initially presented with quotidian fever, leukocytosis, arthralgia, and hyperferritinemia, prompting a broad infectious workup. Extensive testing - including blood and respiratory cultures, gastrointestinal polymerase chain reaction panel (GI PCR), viral serologies (HIV, CMV, EBV), and antigen testing for Legionella, Streptococcus pneumoniae, Ehrlichiosis, Lyme disease, and Cryptococcus - was negative, effectively excluding infectious etiologies and narrowing the differential. The absence of disease-specific biomarkers further complicates recognition, reinforcing the need for clinical judgment and exclusion of mimics [3,8].

The presence of a positive ANA initially introduced diagnostic uncertainty, as ANA positivity is uncommon in AOSD and may raise concern for connective tissue disease overlap. Nonetheless, our patient fulfilled five or more of the Yamaguchi criteria, including two or more major criteria - quotidian fever >39°C for more than one week, arthralgia, leukocytosis with neutrophilia, and negative rheumatoid factor - supporting a diagnosis of AOSD despite ANA positivity. This case highlights that ANA positivity should not preclude the diagnosis when other features strongly align with AOSD and mimics have been systematically excluded.

From a pathophysiologic standpoint, our patient’s striking hyperferritinemia and systemic inflammation reflect the central role of cytokine dysregulation, particularly IL-1β, IL-6, and IL-18, which drive the clinical phenotype and systemic inflammation [4-6]. Increasingly, AOSD is viewed as an autoinflammatory condition with immune overlap, explaining both the heterogeneity of presentations and the occurrence of atypical serologies [7,11].

Management has traditionally relied on corticosteroids and methotrexate, but biologics targeting IL-1 and IL-6 have revolutionized outcomes. Anakinra, canakinumab, and tocilizumab demonstrate high efficacy in refractory disease, improving remission and steroid-sparing rates [2,8,10]. Our patient was started on systemic corticosteroids with a dramatic response, including prompt resolution of fever and associated rash along with arthralgia. Given the systemic phenotype and the need for sustained disease control, therapy was transitioned to the IL-1 receptor antagonist anakinra following discharge, allowing for a gradual taper of systemic corticosteroids. At four-week and 12-week follow-up, the patient demonstrated complete resolution of fever and arthralgia, normalization of inflammatory markers, and no reported adverse effects or disease flares. Management of our patients’ disease course reflects this paradigm shift, where early biologic use optimizes outcomes [7,9,10].

Future advances may come from biomarker integration. Novel candidates such as glycosylated ferritin, IL-18, and S100 proteins could refine diagnosis and prognosis [3,5,9]. Stratifying patients into systemic and articular phenotypes may also tailor therapy [2,7,11]. Collectively, our case supports the use of individualized, biomarker-guided approaches to this complex disorder [9-11].

## Conclusions

This case highlights the diagnostic complexity of AOSD, a rare systemic autoinflammatory disorder that often presents with nonspecific constitutional and inflammatory features. Our patient’s persistent fever, evanescent rash, hyperferritinemia, and systemic involvement in the absence of identifiable infection or malignancy were key in establishing the diagnosis. Early recognition is critical, as delayed diagnosis frequently leads to prolonged empiric antimicrobial use and unnecessary invasive testing. The prompt resolution of symptoms following corticosteroid initiation underscores the importance of considering AOSD in patients with prolonged fever of unknown origin, systemic inflammation, and negative infectious and malignant workup. Clinicians should maintain a high index of suspicion, as timely initiation of immunosuppressive therapy can dramatically improve outcomes.

## References

[1] Gerfaud-Valentin M, Jamilloux Y, Iwaz J, Sève P (2014). Adult-onset Still's disease. Autoimmun Rev.

[2] Kadavath S, Efthimiou P (2015). Adult-onset Still's disease-pathogenesis, clinical manifestations, and new treatment options. Ann Med.

[3] Giacomelli R, Ruscitti P, Shoenfeld Y (2018). A comprehensive review on adult onset Still's disease. J Autoimmun.

[4] Sfriso P, Bindoli S, Galozzi P (2018). Adult-onset Still’s disease: molecular pathophysiology and therapeutic advances. Drugs.

[5] Li S, Zheng S, Tang S, Pan Y, Zhang S, Fang H, Qiao J (2020). Autoinflammatory pathogenesis and targeted therapy for adult-onset Still’s disease. Clin Rev Allergy Immunol.

[6] Rao S, Tsang LS, Zhao M, Shi W, Lu Q (2022). Adult-onset Still's disease: a disease at the crossroad of innate immunity and autoimmunity. Front Med (Lausanne).

[7] Macovei LA, Burlui A, Bratoiu I (2022). Adult-onset Still’s disease-a complex disease, a challenging treatment. Int J Mol Sci.

[8] Tomaras S, Goetzke CC, Kallinich T, Feist E (2021). Adult-onset Still’s disease: clinical aspects and therapeutic approach. J Clin Med.

[9] Bindoli S, Baggio C, Doria A, Sfriso P (2024). Adult-onset Still’s disease (AOSD): advances in understanding pathophysiology, genetics and emerging treatment options. Drugs.

[10] Ma Y, Meng J, Jia J (2021). Current and emerging biological therapy in adult-onset Still's disease. Rheumatology (Oxford).

[11] Castañeda S, Blanco R, González-Gay MA (2016). Adult-onset Still's disease: advances in the treatment. Best Pract Res Clin Rheumatol.

